# Nationwide survey on training and device utilization during tracheal intubation in French intensive care units

**DOI:** 10.1186/s13613-019-0621-9

**Published:** 2020-01-03

**Authors:** M. Martin, P. Decamps, A. Seguin, C. Garret, L. Crosby, O. Zambon, A. F. Miailhe, E. Canet, J. Reignier, J. B. Lascarrou, The Intubation Practices Survey Group, A. Rivière, M. Carles, S. Garnier, H. Dupont, J. Maizel, W. Nicola, A. Mercat, D. Schnell, A. Levrat, V. Cadiergue, P. De Swardt, M. Benhamou, H. Mentec, K. Debbat, R. Sahri, M. Granier, M. H. Hausermann, G. Alvado, J. Pillot, J. Richecoeur, G. Capellier, C. Vinsonneau, J. C. Farkas, L. Favier, L. Feller, Y. Cohen, D. Gruson, A. Vieillard-Baron, O. Michel, E. L’her, N. Pichon, S. Carreira, D. Du Cheyron, K. Chaoui, P. M. Bertrand, M. Attané, M. O. Lafforgue, J. M. Doise, P. Berger, J. M. Thouret, P. Mateu, B. Sauneuf, A. Le Meur, S. De Rudnicki, P. Trouiller, B. Souweine, J. M. Constantin, A. Alvarez, G. Barjon, C. Roth, G. Chevrel, E. Renaud, F. Schortgen, A. Mekontsso Dessap, T. Mayet, J. Rigaud, J. P. Quenot, P. Brofferio, F. Bavozet, S. Beague, H. Vardanyan, O. Delastre, M. Boukhazna, L. Niquet, M. Kaidomar, P. Y. Simonoviez, D. Annane, C. Schwebel, D. Soltani, J. Kempf, E. Delpierre, C. Richard, J. C. Lacherade, A. Herbland, H. Yassine, J. P. Bedos, C. Guitton, A. Sossou, J. Temime, B. Gauche, D. Mathieu, P. Vignon, T. Van der Linden, M. Thyrault, G. Grillet, L. Argaud, C. Pommier, V. Piriou, De Varax, R. Blonde, N Bruder, M. Gainnier, A. Sannini, L. Papazian, M. Leone, E. Cantais, I. Odin, W. Bougouin, M. Bouguettaya, M. Monchi, L. Muller, J. Mariot, A. Sément, J. Roustan, O. Millet, M. Bousta, P. Verdier, K. Klouche, V. Das, M. Lefèvre, P. Linval, K. Kuteifan, P. Y. Bollaert, M. Martin, P. Cocquet, O. Tuil, K. Koubi, L. Muller, P. Hazera, E. Couadau, T. Boulain, S. Mons, J. F. Timsit, C. Bruel, J. L. Diehl, B. Mégarbane, A. Combes, E. Azoulay, T. Similowski, J. P. Mira, M. Fartoukh, B. Guidet, W. Picard, O. Barbot, H. Outin, R. Robert, J. L. Dubost, M. Fejjal, M. Moriconi, W. Bouguoin, B. Mourvillier, Y. Le Tulzo, P. Beuret, A. Delahaye, P. Herbecq, F. Tamion, G. Dardenne, B. Letellier, O. Martinet, L. Popoff, F. Zeni, M. Ramakers, L. Muller, J. Hoff, C. Galland, V. Boisson, J. F. Vincent, A. Mofredj, P. Ubrich, D. Gizolme, F. Meziani, F. Schneider, T. Dulac, M. Bemer, T. Seguin, B. Riu, O. Leroy, P. F. Dequin, G. Simon, Jeannot, F. Lambiotte, B. Levy, J. Huntzinger, C. Floriot, R. Ravan, F. Blot, S. Le Liron Manzon

**Affiliations:** Service de Médecine Intensive Réanimation, Centre Hospitalier Universitaire de Nantes, 44093 Nantes Cedex 9, France

**Keywords:** Survey, Airway, Equipment, Training, Videolaryngoscope, Intensive care unit, Endotracheal intubation

## Abstract

**Background:**

Intubation is a lifesaving procedure that is often performed in intensive care unit (ICU) patients, but leads to serious adverse events in 20–40% of cases. Recent trials aimed to provide guidance about which medications, devices, and modalities maximize patient safety. Videolaryngoscopes are being offered in an increasing range of options and used in broadening indications (from difficult to unremarkable intubation). The objective of this study was to describe intubation practices and device availability in French ICUs.

**Materials and methods:**

We conducted an online nationwide survey by emailing an anonymous 26-item questionnaire to physicians in French ICUs. A single questionnaire was sent to either the head or the intubation expert at each ICU.

**Results:**

Of 257 ICUs, 180 (70%) returned the completed questionnaire. The results showed that 43% of intubators were not fully proficient in intubation; among them, 18.8% had no intubation training or had received only basic training (lectures and observation at the bedside). Among the participating ICUs, 94.4% had a difficult intubation trolley, 74.5% an intubation protocol, 92.2% a capnography device (used routinely to check tube position in 69.3% of ICUs having the device), 91.6% a laryngeal mask, 97.2% front-of-neck access capabilities, and 76.6% a videolaryngoscope. In case of difficult intubation, 85.6% of ICUs used a bougie (154/180) and 7.8% switched to a videolaryngoscope (14/180). Use of a videolaryngoscope was reserved for difficult intubation in 84% of ICUs (154/180). Having a videolaryngoscope was significantly associated with having an intubation protocol (*P *= 0.043) and using capnography (*P *= 0.02). Airtraq^®^ was the most often used videolaryngoscope (39.3%), followed by McGrath^®^Mac (36.9%) then by Glidescope^®^ (14.5%).

**Conclusion:**

Nearly half the intubators in French ICUs are not fully proficient with OTI. Access to modern training methods such as simulation is inadequate. Most ICUs own a videolaryngoscope, but reserve it for difficult intubations.

## Introduction

Many factors contribute to the considerable morbidity and mortality associated with orotracheal intubation (OTI) in critically ill patients [1]. The patients are highly vulnerable, due in particular to the presence of organ failures and absence of fasting. In addition, OTI is often performed by junior physicians who are not yet fully proficient with the procedure. Thus, emergency intubation in the ICU is more often difficult than is scheduled intubation in the operating room, and complications develop in up to half the cases [2, 3]. Up to a fourth of OTIs in the ICU are associated with severe complications [4, 5], including cardiac arrest [6].

Several measures are recommended to minimize the risk of complications of OTI [7, 8]. Adverse effects of anesthetic agents and positive pressure ventilation can be minimized by volume repletion, vasoactive drugs, the use of short-acting anesthetics, and/or preoxygenation with noninvasive ventilation [9, 10]. OTI conditions should be optimized by careful attention to patient installation, prediction of difficult intubation based on the MACOCHA score [2], the application of appropriate algorithms [8], neuromuscular blockade, and the routine use of capnography to check endotracheal tube position. Combining these measures within a routinely applied protocol decreases the frequency of severe complications [1].

One of the predictors of complication-free OTI, however, is success at the first attempt [11, 12]. Consequently, maximizing the likelihood of first-pass success deserves every effort. Various devices and techniques have been developed to increase the first-pass success rate. The devices include metallic blades [13], stylets and bougies [14], and the more recently introduced videolaryngoscope, which improves visualization of the glottis and allows a second operator to provide assistance. However, in a randomized controlled trial reported in 2017 [15] and subsequent meta-analysis [16], the routine use of a videolaryngoscope in ICU patients failed to produce higher first-pass success rates compared to the Macintosh laryngoscope and was associated with a higher risk of serious adverse events. The 2018 guidelines issued by the Difficult Airway Society [8] recommend that a videolaryngoscope should be available in all ICUs and should be considered for the first attempt when laryngoscopy is predicted to be difficult [17]. Videolaryngoscopy is increasing in popularity despite persistent uncertainties about indications and benefits. A videolaryngoscope was available in 28% of French ICUs in 2013 [18] and 50% of British ICUs in 2017 [19]. Both the range of videolaryngoscope options and the indications of videolaryngoscopy are expanding. However, benefits from videolaryngoscopy may occur only in patients with difficult airways and may vary with the type of videolaryngoscope used [8, 15]. Further studies of the effects of videolaryngoscopy according to patient characteristics and type of device used are clearly needed. Finally, another approach to improving patient safety during OTI consists in maintaining oxygenation in the event of unanticipated difficulties and failed facemask ventilation. This goal can be achieved using supraglottic airway devices (SADs), preoxygenation, apneic oxygenation or, as a last resort, emergency front-of-neck access (FONA) [8].

Any further study aimed at optimizing patient safety during OTI in the ICU would occur against a backdrop of considerable heterogeneity in OTI practices, equipment, and training. This heterogeneity would have to be factored into the study design and the interpretation of the results. Therefore, detailed knowledge of practices is needed.

The objective of this nationwide questionnaire survey was to describe intubation practices and equipment in ICUs in France, with special attention to the proportion of ICUs equipped with at least one videolaryngoscope in 2019. The main hypothesis was that a videolaryngoscope was available in more than half of French ICUs.

## Materials and methods

### Study design

An online anonymous survey was conducted by having two study investigators (MM and JBL) email a 26-item questionnaire to all 256 medical and medical–surgical ICUs identified during the latest available survey of ICUs in France [20]. The first version of the questionnaire was built by the two study investigators adapted from [19], tested and modified by each, and finally tested and modified by three intensivists (ALM, JH, and JCL, from the Intubation Practices Survey group). The questionnaire was established using Google Forms (Google, Mountain View, CA) (eSupplement). Each ICU received a single questionnaire, which was sent either to the head or to the airway management expert of the ICU. A reminder was sent to nonrespondents after 14 days. When there was no response to this second email, the ICU office was contacted by phone to obtain the name and email address of the airway management expert, who was then sent the questionnaire, or to ask the secretary to deliver the questionnaire to this expert; a reminder was sent to nonrespondents after 14 days. The survey was conducted from April 2019 to June 2019. The two study investigators manually checked the completed questionnaires to ensure that a single questionnaire was used for each ICU.

### Questionnaire (eSupplement)

The questionnaire collected information about global airway management, staff expertise, awareness of expert recommendations, and availability and use of videolaryngoscopy (Additional file 1: Questionnaire). Several devices that use digital or optical imaging to facilitate tracheal intubation were listed in the questionnaire. These devices were chosen pragmatically, and space was available on the questionnaire to report the use of devices that were not listed. Six videolaryngoscopes were specifically named in the questionnaire: Airtraq^®^ (Prodol Meditec, Guecho, Spain) in its optical-only and video-camera versions, C-MAC^®^ (Karl Storz, Slough, UK) with no details about type of blade (standard or D), GlideScope^®^ (Verathon, North Creek Parkway Bothell, WA), King Vision^®^ (Ambu, St Ives, UK), McGrath^®^ Mac (Aircraft Medical, Edinburgh, UK), and APA™ (Venner Medical, Dänischenhager, Germany). Experienced intubators were defined as intensivists who had either worked in ICUs for at least 5 years or worked in ICUs for at least 1 year after receiving at least 2 years of anesthesiology training [21].

### Primary and secondary objectives

The primary objective was to evaluate the proportion of ICUs equipped with at least one videolaryngoscope.

The secondary objectives were to evaluate the use of videolaryngoscopy; the characteristics of intubators in ICUs, including knowledge of airway control guidelines; and potential associations between those characteristics and availability of a videolaryngoscope.

### Statistics

Qualitative variables were described as *n* (%) and quantitative variables as mean ± SD if normally distributed and median [25th–75th percentiles] otherwise. Qualitative variables were compared across groups using Fisher’s exact test. To compare quantitative variables across groups, we applied Student’s *t* test. Missing data were disregarded. *P* values smaller than 0.05 were considered significant. All statistical analyses were performed using STATA version 13 (STATA Corp, College Station, TX).

## Results

Of the 256 French medical and medical–surgical ICUs invited to participate in the survey, 180 (70%) returned completed questionnaires (Additional file 2: Figure S1).

### Geographic and demographic data

**Fig. 1:**
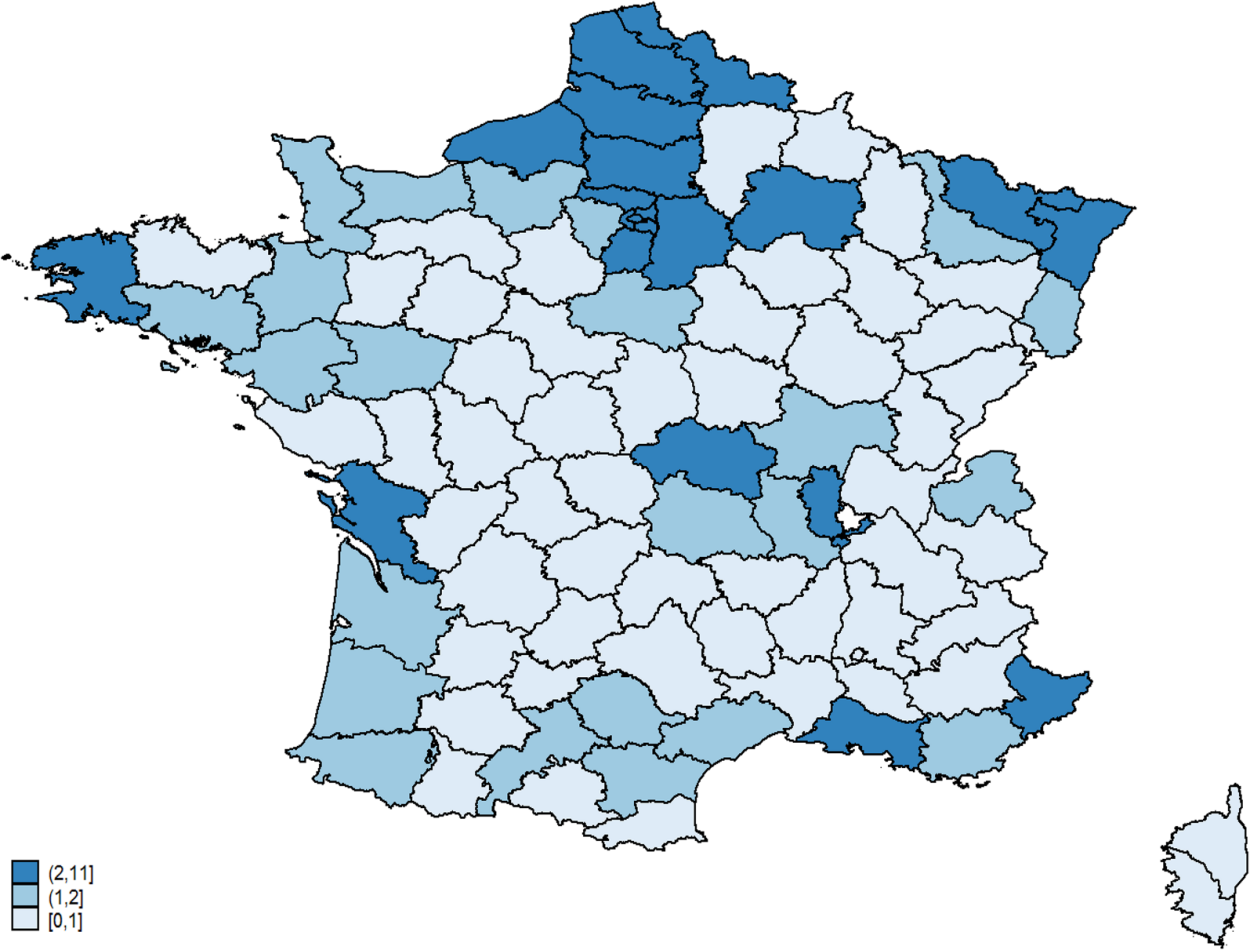
Distribution of the 180 surveyed ICUs in continental France

Figure 1 shows that the participating ICUs were evenly distributed across France, with completed questionnaires received from 86 of the 101 departments of the country. The distribution of hospital types was as follows: university hospitals, 113/180 (63%); district general hospitals, 50/180 (28%), and community hospitals, 17/180 (9%). Of the 180 participating ICUs, 139 (77.3%) admitted both medical and surgical patients and 41 (21.7%) only medical patients. The median annual number of admissions was 600 [405–800] and the median annual number of OTIs was 180 [100–300].

### Orotracheal intubation (OTI) skill levels and training

The median number of experienced intubators per ICU was 6 [5–8] and the median number of trainee intubators per ICU was 5 [3–8]. The total numbers of experienced and trainee intubators in the 180 participating ICUs were 1270 and 974, respectively.

**Fig. 2:**
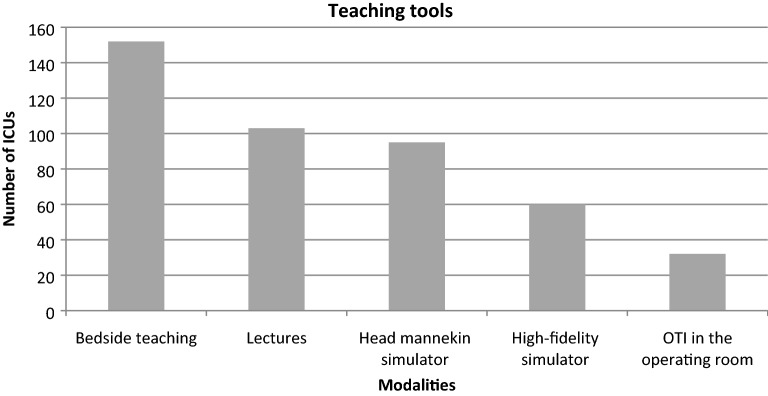
Distribution of methods used to teach orotracheal intubation (OTI) skills

Of the 180 ICUs, 174 (96.6%) reported using the following methods to provide OTI training: teaching at the bedside, 152/174 (87.3%); lectures, 103/174 (59.2%); head manikin training, 95/174 (54.6%); high-fidelity simulators, 60/174 (34.5%); and OTI in the operating room, 32/174 (18.4%) (Fig. 2). Of the 174 ICUs, 21 (12.1%) used only bedside teaching and 50 (28.7%) only bedside teaching and lectures. A manikin simulator and/or a high-fidelity simulator was used in 105/174 (60.3%) ICUs. Of the 180 participating ICUs, 142 (78.9%) had easy access to a manikin head for OTI training within their institution, but 37 (26.1%) of them did not use manikin simulation for OTI training. High-fidelity simulation was more often available at university hospitals (28/113) and district general hospitals (28/50) than in community hospitals (4/17) (*P *< 0.001).

### Devices and tools

Of the 180 participating ICUs, 170 (94.4%) had a difficult OTI trolley and 134 (74.4%) had a specific OTI protocol. Furthermore, 166 ICUs (92.2%) had capnography equipment, although only 115/166 (69.3%) used capnography routinely to check endotracheal tube position (i.e., 115/180 [64%] of all participating ICUs). Having capnography equipment was not significantly associated with having an OTI protocol (10 of 14 ICUs without capnography vs. 124 of 166 with capnography had an OTI protocol; *P *= 0.07). Neither was a significant association found between routine use of capnography and having an OTI protocol (43 of 65 ICUs that did not vs. 91 of 115 that did use capnography routinely had an OTI protocol, *P *= 0.07).

The OTI method used for the first attempt in patients with a difficult or unremarkable airway was Macintosh laryngoscopy alone in 150/180 (83.3%) ICUs; a stylet or bougie with Macintosh laryngoscopy in 16 (8.9%) and 6 (3.3%) ICUs, respectively; a videolaryngoscope in 6 (3.3%) ICUs; and a videolaryngoscope with a stylet in 2 (1.1%) ICUs. When intubation proved difficult despite good visualization of the glottis, a bougie was used in 154/180 (85.6%) ICUs, a videolaryngoscope in 14/180 (7.8%) ICUs, and a stylet in 12/180 ICUs (6.6%).

Of the 180 participating ICUs, 165 (91.6%) had a laryngeal mask.

### Knowledge of guidelines for airway management in ICU patients

Of the 180 respondents, 167 (92.7%) reported being aware of French guidelines [7] and 42 (23.3%) of guidelines from other countries (UK [8], India [22]). Finally, 12 (6.6%) of the 180 respondents reported having no knowledge of any intubation guidelines.

### Availability of a videolaryngoscope

A videolaryngoscope was available in 138/180 (76.6%) ICUs and could be obtained by five additional ICUs from an operating room, yielding a total of 143/180 (79.4%). Availability of a videolaryngoscope did not differ significantly across hospital types (university hospitals, 39/50, 78%; district general hospitals, 88/113, 77.8%; and community hospitals, 11/17, 64.7%) (*P *= 0.47).

Having a videolaryngoscope in the ICU was significantly associated with having an OTI protocol (26 of 42 ICUs without vs. 108 of 138 ICUs with a videolaryngoscope had an OTI protocol, *P *= 0.04), having capnography equipment (35/42 vs. 131/138, *P *= 0.02), and routinely using capnography (18/42 vs. 97/139, *P *= 0.002). In contrast, having a videolaryngoscope in the ICU was not significantly associated with having a difficult intubation trolley (40/42 vs. 130/138; *P *= 0.99).

**Fig. 3:**
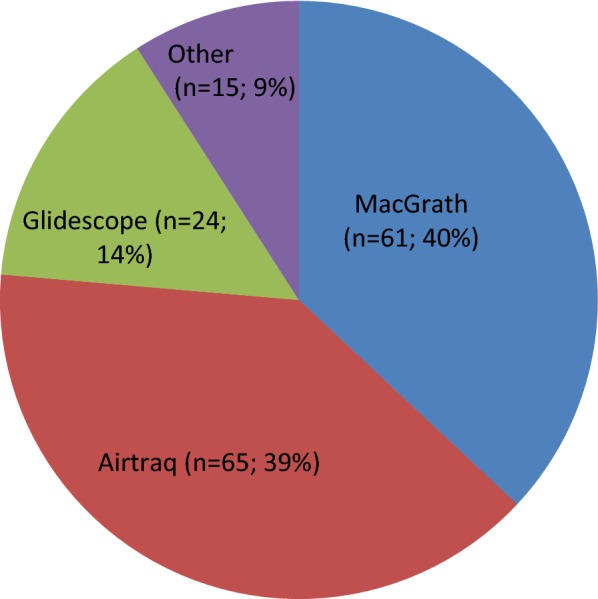
Distribution of videolaryngoscope models available in 138 of the 180 surveyed ICUs

Of the 138 ICUs with at least one videolaryngoscope, 65 had an Airtraq^®^ (53 in the optic fiber version and 12 in the video version), 61 had a McGrath^®^Mac, and 24 had a GlideScope^®^. Only 7 ICUs had a King Vision^®^, 3 an UEScope^®^, 2 a CMAC^®^, and 2 a Pentax AWS^®^; the device was not specified for 1 ICU (Fig. 3). The total exceeds 138 because 24 ICUs had more than one videolaryngoscope: 21 had two models and 3 had three models, yielding a total of 165. The most common combinations of models were Airtraq^®^ plus McGrath^®^Mac (13/24) and Airtraq^®^ plus GlideScope^®^ (5/24), indicating that ICUs tended to choose one device with and another without an operating channel (20/24 ICUs).

Of the 138 ICUs with at least one videolaryngoscope, 93 (67.4%) had acquired the device within the past 5 years (including 17 [17/138, 12.3%] within the past year). On the other hand, 42/138 (30.4%) ICUs had been using their videolaryngoscope for more than 5 years (date of purchase was not specified for three ICUs).

### Use of videolaryngoscopy

Of the 138 ICUs with at least one videolaryngoscope, 22 (15.9%) used the device often or routinely and 116 (84%) reserved the use of the device for predicted difficult intubations. Only 8 (4.4%) ICUs reported routinely using a videolaryngoscope for the first attempt, including 2 (1.1%) ICUs that used the videolaryngoscope with a stylet.

### Reasons for videolaryngoscopy unavailability

The 42 ICUs without a videolaryngoscope reported the following reasons for not having acquired the device: insufficient funds, *n* = 15 (35.7%); insufficient evidence of benefits, *n* = 14 (33.3%); availability of a videolaryngoscope from an operating room, *n* = 5 (11.9%); purchase currently under consideration, *n* = 4 (9.5%); no awareness of the existence of videolaryngoscopes, *n* = 1; other higher priority, *n* = 11; and belief that fiberoptic intubation is sufficient, *n* = 2.

### Cannot intubate, cannot oxygenate (CICO) situation

For the cannot intubate, cannot oxygenate (CICO) situation, emergency front-of-neck access (FONA) was available in 175 (97.2%) of the 180 ICUs. The main reported FONA technique was cricothyroidotomy, usually with a cricothyroidotomy catheter set (118/175, 67.4%) and less often by surgical cricothyroidotomy with a scalpel and bougie (26/175, 14.8%). Percutaneous transtracheal jet ventilation was available in 32 ICUs (Enk^**®**^, 28/175 [16%] or Manujet^**®**^, 4/175 [2.3%]).

## Discussion

Our survey with a high response rate of 70% draws a detailed picture of OTI practices, particularly regarding junior physician training and the availability and use of videolaryngoscopes, in French ICUs. The vast majority of ICUs used Macintosh laryngoscopy for first-attempt OTI in patients with an unremarkable or difficult airway. Nevertheless, most ICUs had at least one videolaryngoscope, whose use was generally reserved for difficult OTIs. Having a videolaryngoscope was associated with having a written OTI protocol and using capnography routinely to assess tube position. Training of intubators was often insufficient.

### Training methods

All but 6 of the 180 participating ICUs provided OTI training to junior physicians. Of the 2244 physicians performing OTIs in the surveyed ICUs, 974 (43%) were OTI novices and among them 185 (19%) received either no training or only basic training consisting of lectures and observation with or without supervised OTI at the bedside. Most scientific societies recommend initial theoretical training followed by practice on manikins and high-fidelity simulators then by supervised OTI in patients until proficiency is achieved, after which further practice is needed for skill maintenance [7]. A meta-analysis showed that, compared to other training methods (lectures, self-study, videos, and operating room training), simulation training using low- or high-fidelity tools was associated with better patient outcomes, learner satisfaction, and skills, although not with better knowledge [23]. Of the 174 ICUs in our survey that provided OTI training, 105 (60.3%) used some form of simulation and 60 (34.5%) high-fidelity simulators. Proficiency in airway management must be acquired and maintained by regular practice of all the techniques involved, which would not be expected to occur via clinical practice alone. Consequently, access to low- and high-fidelity simulators must be improved. Nonetheless, the availability of OTI simulators in French ICUs does not seem to have increased in recent years [18]. Funding is the main obstacle to the expansion of simulation training. Conceivably, OTI training hubs could be established to ensure the intensive use of each simulator, thereby decreasing costs by minimizing the number of simulators needed.

### Global airway management

A difficult intubation trolley was available in 94% of the surveyed ICUs, in keeping with the 97% proportion in a 2013 French survey, indicating good compliance with guidelines on this point. The 4th National Audit Project (NAP_4_) conducted by the Royal College of Anaesthetists and the Difficult Airway Society [5] found that delays in obtaining the necessary airway-management equipment were common, particularly in difficult situations when intubator performance was potentially impaired by cognitive overload, time pressure, and stress [24]. However, according to an earlier survey, some intensivists were unaware of the location of the trolley [18].

Capnography equipment was available in nearly all (92%) the surveyed ICUs. Among these ICUs, however, only 69% routinely used capnography to check endotracheal tube position. The use of capnography is now recommended as the best means of confirming intratracheal placement of an artificial airway [8].

Only three-fourths of the surveyed ICUs had an OTI protocol. One possible explanation to this finding is that an OTI protocol is only a grade 2+ recommendations in French guidelines [7]. In addition, the usefulness of a checklist was challenged recently [25].

### Methods used to visualize the glottis

Macintosh laryngoscopy was used for the first OTI attempt in 83% of surveyed ICUs. A stylet or bougie was used with the Macintosh laryngoscope for the first attempt in 16 (8.9%) and 6 (3.3%) ICUs, respectively. In a randomized trial reported in 2018, the use of a bougie produced a significantly higher first-pass success rate compared to an endotracheal tube with a stylet in patients undergoing emergency OTI [14]. Consistent with this finding, 154 (85.6%) of the ICUs in our survey used a bougie when intubation was difficult despite good visualization of the glottis. The 2017 French guidelines recommend use of a bougie for the first attempt only for patients whose MACOCHA score is 3 or higher. However, in our study, 14 (7.8%) ICUs switched to a videolaryngoscope in the event of difficult intubation, irrespective of the MACOCHA score.

#### Videolaryngoscope availability

Most ICUs had at least one videolaryngoscope, with no significant differences across type of hospital. The device had usually been acquired within the past 5 years, reflecting the current high level of interest in videolaryngoscopy. Whether this enthusiasm for videolaryngoscopy is supported by the scientific evidence deserves discussion [17]. The many reported benefits of videolaryngoscopy include improved visualization of the glottis [26], a reduction in applied force [27], a short learning curve [28], and improved training of novices [29]. However, the effect on patient outcomes, which is the key indicator of benefits, remains unclear. In addition, the availability of various types of videolaryngoscope, each of which may have specific effects, is a complicating factor. The Aitraq^®^ and McGrath^®^Mac devices accounted for three-quarters of the videolaryngoscopes in the surveyed ICUs, whereas the GlideScope^®^ was less popular. In the UK, Airtraq^®^ is also the most widely used laryngoscope [19].

For OTI in the operating room, compared to a conventional laryngoscope, the Airtraq^®^ was associated with a significantly lower risk of first-pass failure, a higher proportion of patients with Cormack–Lehane grade 1 visualization, a shorter time to successful OTI, and lower rates of oropharyngeal complications [30]. Results with other videolaryngoscopes were similarly promising, with improved glottis visualization translating into lower first-pass failure rates or shorter times to successful OTI in the operating room [31]. For Airtraq^®^, the availability of a disposable option may result in cost savings when reserved for specific situations and may decrease the risk of infection. Finally, in a manikin study the Airtraq^®^ had a better learning curve compared to the McGrath^®^Mac and GlideScope^®^ [32].

The McGrath^®^Mac was nearly as popular as the AirTraq^®^ in the surveyed ICUs. This device was found to be promising in a before/after study in French ICUs [33]. The Macintosh-like angulation of the blade allows use for either direct or indirect glottis visualization. This feature may be useful in some situations, for instance for patients with abundant secretions [34]. Moreover, this videolaryngoscope and its consumables are less expensive compared to other videolaryngoscopes.

The Glidescope^®^ was chosen by about one-sixth of the surveyed ICUs, a proportion similar to that reported in the UK [19]. This device performed well in an observational study [35]. The hyperangulated blade suggests that its usefulness may be greatest in patients with difficult airways. Among ICUs that had more than one videolaryngoscope in our study, two-fifths had a GlideScope^®^ and another device with a less sharply angulated blade. The GlideScope^®^ allows visualization on a distant screen, thus potentially facilitating supervision and assistance.

Among ICUs that used videolaryngoscopy, 14% had more than one videolaryngoscope. In four-fifths of cases, one device had an operating channel and the other did not, providing two options for improving glottic catheterization.

The most commonly reported reasons for not having a videolaryngoscope were lack of funds and lack of convincing evidence of benefits.

#### Indications of videolaryngoscopy

Of the 138 ICUs with at least one videolaryngoscope, only 8 reported routinely using a videolaryngoscope for the first OTI attempt and 14 others used videolaryngoscopy frequently. Videolaryngoscopy for the first OTI attempt may be best reserved for patients whose MACOCHA score is 3 or higher [7] or who meet at least two criteria for difficult OTI [7]. In a randomized trial of unselected ICU patients, first-pass success was not significantly more common with a videolaryngoscope after adjustment on the MACOCHA score [15]. The restrictions placed on the use of videolaryngoscopy are likely to limit clinical training opportunities, further supporting the need for achieving widespread availability of simulation training.

### Cannot intubate, cannot oxygenate (CICO) situation

Most of the surveyed ICUs had a supra-glottic airway device for oxygenation and the ability to perform emergency FONA. Needle cricothyroidotomy was by far the preferred FONA technique, with only a minority of ICUs performing surgical cricothyroidotomy. However, the 2015 Difficult Airway Society (DAS) guidelines recommend surgical cricothyroidotomy using a scalpel, bougie, and tube for emergency FONA [8]. The NAP_4_ report on airway complications during anesthesia, in the ICU, and in the emergency room showed that needle cricothyroidotomy was associated with high rates of complications and failure due to insufficient operator proficiency, lack of specific equipment, and/or catheter kinking, malposition, or displacement [5]. In addition, a metanalysis [32] and observational data collected via a smartphone application showed higher success rates with surgical vs needle cricothyroidotomy [36].

### Study limitations

The main limitation is inherent in the survey design, which involved collecting the data via a questionnaire completed by a single intensivist in each ICU. However, the high response rate of 70% is a major strength. Nevertheless, selection bias may have occurred, with nonresponding ICUs being more likely to be nonusers of videolaryngoscopy, resulting in overestimation in our survey of the proportion of ICUs equipped with at least one videolaryngoscope. We chose not to include surgical ICUs, as surgical ICUs are staffed by anesthetists with extensive OTI experience. A single questionnaire was completed in each ICU, in theory by the head of the unit or the airway management expert, who may not have accurately evaluated OTI practices. Insufficient knowledge may have been greatest in ICUs without an OTI protocol. In addition, we collected data on the numbers of each category of intubator in each ICU but not on the numbers of OTIs performed by each category. The survey questions were not developed using Delphi rounds. However, they were chosen by two highly experienced intensivists then evaluated and modified by three airway management experts.

## Conclusion

This survey depicts OTI practices in ICUs throughout France in the era of videolaryngoscopy. Overall, practices were consistent with recent guidelines. Access to modern simulators for OTI training was inadequate. Most ICUs were equipped to perform videolaryngoscopy, but reserved this method for difficult OTI. Macintosh laryngoscopy was by far the most widely used first-attempt method.

## Supplementary information

**Additional file 1: Document S1.** Questionnaire.

**Additional file 2: Figure S1.** Visual abstract.

## Data Availability

The dataset is available on reasonable request to the corresponding author.
