# Supplementary material for: Nationwide survey on training and device utilization during tracheal intubation in French intensive care units
Source: Ann Intensive Care. 2020 Jan 3;10:2. doi: 10.1186/s13613-019-0621-9 (PMC6942097; doi:10.1186/s13613-019-0621-9)
Supplement: Supplementary file 1 — Additional file 1: Document S1. Questionnaire. [file 13613_2019_621_MOESM1_ESM.pdf]

# Évaluation des pratiques pour l'intubation en réanimation en France

Chers confrères,

L'intubation trachéale reste associée à une morbidité importante en réanimation. Si l'intérêt des vidéolaryngoscopes est désormais reconnu dans le cadre de l'intubation difficile, son utilisation systématique présente à ce jour un niveau de preuve insuffisant avec une littérature contradictoire. Les éléments divergents pouvant influencer les résultats des études sur le sujet portent essentiellement sur l'expérience des opérateurs et leur formation, le type de vidéolaryngoscope testé, et les éléments d'aide à la cathétérisation glottique utilisés (guide , canal opérateur...).

Il nous semble donc important de réaliser une enquête de pratique nationale sur l'intubation en réanimation.

Nous vous convions donc à répondre à un bref questionnaire concernant vos pratiques d'intubation dans votre unité de réanimation.

*Obligatoire

1. **Quel est le nom de votre hôpital** *

   ____________________

2. **Quel est la ville de votre hôpital** *

   ____________________

3. **Quel est le nom de votre chef de service** *

   ____________________

4. **Votre réanimation est elle?**

   *Une seule réponse possible.*

   - ◯ Chirurgicale
   - ◯ Médicale (MIR)
   - ◯ Polyvalente
   - ◯ Spécialisée (chirurgie cardiaque, neurochirurgie...)

5. **Combien d'admissions réalisez vous en moyenne chaque année dans votre réanimation? (hors USC)**

   ____________________

6. **Combien d'intubations sont réalisées approximativement chaque année dans votre service ?** *

   ____________________

7. **Combien il y a-t-il de médecin "expert" en intubation dans votre service ? (expert = médecin ayant plus de 5 ans d'activité en réanimation ; ou plus d'1 an d'activité de réanimation s'il a reçu une formation préalable de 2 ans en anesthésie; pour les internes DESAR en fin de cursus, 4 semestres d'anesthésie et 2 semestres de réanimation validés)**

_______________

8. **Combien il y a-t-il de médecin "non expert" en intubation dans votre service ? (internes compris)** *

_______________

9. **Formez-vous vos médecins non-experts à l'intubation ?** *
*Une seule réponse possible.*
   - ( ) Oui
   - ( ) Non

10. **Si oui, comment ?** *
*Plusieurs réponses possibles.*
   - [ ] Apprentissage théorique (cours magistral)
   - [ ] Apprentissage au lit du malade
   - [ ] Intubation sur tête de mannequin
   - [ ] Atelier de simulation
   - [ ] Autre : _______________

11. **Avez vous la possibilité, au sein de votre établissement, d'accéder à une tête d'intubation et/ou un mannequin pour simuler une intubation?** *
*Une seule réponse possible.*
   - ( ) oui
   - ( ) non

12. **Existe-t-il un protocole d'intubation dans votre unité?** *
*Une seule réponse possible.*
   - ( ) oui
   - ( ) non

13. **Votre service est-il doté d'un capnogramme?** *
*Une seule réponse possible.*
   - ( ) oui
   - ( ) non

14. **Son utilisation est-elle systématique en cas d'intubation ?** *
*Une seule réponse possible.*

- oui
- non

15. **Quel est la technique première utilisée pour l'intubation** *
*Une seule réponse possible.*

- Laryngoscope de Macintosh sans mandrin
- Laryngoscope de Macintosh avec mandrin court ("stylet")
- Laryngoscope de Macintosh avec mandrin long béquillé ("bougie" ou "FROVA")
- Vidéolaryngoscope sans mandrin
- Vidéolaryngoscope avec mandrin court ("stylet")
- Vidéolaryngoscope avec mandrin long béquillé ("bougie" ou "FROVA")

16. **En cas de difficulté à catéthériser la glotte malgré une vision glottique correcte, qu'utilisez vous en premier recours** *
*Une seule réponse possible.*

- Un mandrin court
- Un mandrin long béquillé
- Un vidéolaryngoscope

17. **Votre service est-il doté d'un vidéolaryngoscope ?** *
*Une seule réponse possible.*

- Oui
- Non *Passez à la question 21.*

## Vidéolaryngoscope

18. **Quel est ou quels sont le (les) vidéolaryngoscope(s) dont vous disposez (attention, il doit être situé dans le service et non au bloc opératoire) ?**
*Plusieurs réponses possibles.*

- [ ] MacGrath
- [ ] APA
- [ ] Glidescope
- [ ] Airtraq - version fibre optique
- [ ] Airtraq - version vidéo (caméra)
- [ ] C-MAC
- [ ] KingVision
- [ ] Autre : ____________________

19. **Si oui, depuis combien de temps ? (si vous possédez plusieurs vidéolaryngoscopes, délai d'acquisition du plus ancien)** *
*Une seule réponse possible.*

- < 1an
- 1-5 ans
- > 5 ans

20. **Quel usage en faite vous?** *
*Une seule réponse possible.*

- Utilisation régulière voir systématique
- Utilisation réservée aux situations d'intubation difficile ou prévue difficile.

*Passez à la question 22.*

## Absence de vidéolaryngoscope

21. **Si votre service ne possède pas de vidéolaryngoscope, est-ce :** *
*Une seule réponse possible.*

- par choix (littérature non concluante)
- par manque de budget
- par ignorance
- Autre : ____________________

## Intubation difficile

22. **Disposez-vous d'un masque laryngé ?** *
*Une seule réponse possible.*

- Oui
- Non

23. **Disposez-vous d'un chariot d'intubation difficile dédié (ou d'un tiroir spécifique dédié)?** *
*Une seule réponse possible.*

- Oui
- Non

24. **Avez-vous connaissance des recommandations existantes en réanimation ?** *
*Plusieurs réponses possibles.*

- [ ] RFE SFAR/SRLF2016 sur intubation et extubation du patient de réanimation: Quintard H Anaesth Crit Care Pain Med. 2017 Oct;36(5):327-341
- [ ] Recommandation anglaise sur l'intubation du patient en état critique: Higgs A Br J Anaesth. 2018 Feb;120(2):323-352
- [ ] Recommandations indiennes sur l'intubation en réanimation: Myatra HN Indian J Anaesth. 2016 Dec;60(12):922-930
- [ ] Aucune des 3

25. **En cas de scénario: "intubation & ventilation impossible" ou "CICO: can't intubate & can't ventilate", disposez-vous d'un kit d'abord trachéal d'urgence ?** *

*Une seule réponse possible.*

- Oui
- Non *Après avoir répondu à la dernière question de cette section, cessez de remplir ce formulaire.*

26. **De quel type ?** *

*Une seule réponse possible.*

- Crico-thyroidotomie par scapel et mandrin long béquillé
- Crico-thyroidotomie non chirurgicale par dispositif dédié
- Ventilation haute pression: Manujet (VBM Medical)
- Dispositif spécifique: Enk (Cook Medical)
- Dispositif spécifique:Ventrain (Ventino Medical)

---

Fourni par
Google Forms
