# Supplementary figures and images for: Nationwide survey on training and device utilization during tracheal intubation in French intensive care units
Source: Ann Intensive Care. 2020 Jan 3;10:2. doi: 10.1186/s13613-019-0621-9 (PMC6942097; doi:10.1186/s13613-019-0621-9)

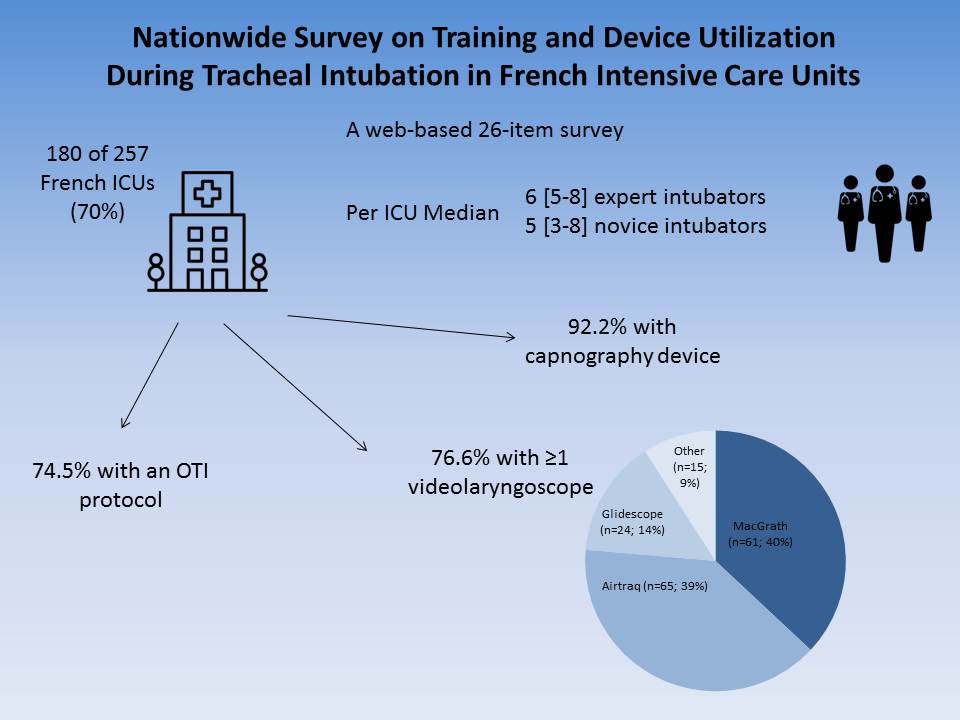

Supplement: Supplementary file 2 — Additional file 2: Figure S1. Visual abstract. [file 13613_2019_621_MOESM2_ESM.jpg]
